# Handling Uncertainty in Models of Seismic and Postseismic Hazards: Toward Robust Methods and Resilient Societies

**DOI:** 10.1111/risa.13663

**Published:** 2020-12-24

**Authors:** Brian H. MacGillivray

**Keywords:** Earthquake hazards, model uncertainty, risk assessment

## Abstract

Earthquakes, tsunamis, and landslides take a devastating toll on human lives, critical infrastructure, and ecosystems. Harnessing the predictive capacities of hazard models is key to transitioning from reactive approaches to disaster management toward building resilient societies, yet the knowledge that these models produce involves multiple uncertainties. The failure to properly account for these uncertainties has at times had important implications, from the flawed safety measures at the Fukushima power plant, to the reliance on short‐term earthquake prediction models (reportedly at the expense of mitigation efforts) in modern China. This article provides an overview of methods for handling uncertainty in probabilistic seismic hazard assessment, tsunami hazard analysis, and debris flow modeling, considering best practices and areas for improvement. It covers sensitivity analysis, structured approaches to expert elicitation, methods for characterizing structural uncertainty (e.g., ensembles and logic trees), and the value of formal decision‐analytic frameworks even in situations of deep uncertainty.

## 1 HANDLING UNCERTAINTY IN MODELS OF EARTHQUAKE‐INDUCED HAZARDS: CHALLENGES AND DEFINITIONS

Earthquakes, tsunamis, and landslides place a devastating toll on human lives, critical infrastructure, and ecosystems, particularly in “developing” nations where major population centers are often clustered in areas of high seismicity and exposed coastal regions (Hill, Sparks, & Rougier, 2013). Processes of global change are combining to exacerbate the risks posed by these geophysical events: hillside deforestation together with climate change is expected to increase landslide activity (Crozier, 2010); land‐use changes and modification of the nearshore environment have broadened areas at risk from tsunami inundation (Alongi, 2008; Titov et al., 2011); rapid, unplanned urbanization has led to many cities being ill‐equipped to withstand major earthquakes (Smith, 2013); while the widespread displacement of traditional institutions and ecological knowledge has eroded adaptive capacities in many regions (Lauer et al., 2013). Harnessing the predictive capabilities of hazard models is key to transitioning from reactive approaches to disaster management toward building resilient societies, alleviating poverty, and driving sustainable growth. Yet, while formal models play an increasing role in characterizing hazards and exploring mitigation options, the knowledge that they produce involves multiple sources of uncertainty. Widespread nonlinearities make results highly sensitive to initial and boundary conditions that are themselves often poorly understood; parameter values are only partially constrained by theory or empirics; and model structures contain significant omissions and idealizations whose implications are challenging to evaluate (Iverson, 2003; Oreskes, Shrader‐Frechette, & Belitz, 1994). Of course, as Box (1979) observed, all models are wrong, but some are useful, meaning that the above challenges are by no means grounds to reject the value of such models. The point instead is that hazard models of earthquakes, landslides, and tsunamis involve multiple, nontrivial sources of uncertainty, meaning that statistical techniques that focus on random error are insufficient on their own (e.g., local sensitivity analysis, confidence intervals, *p*‐values) (c.f. Aven, 2013a; Cox, 2012; Greenland, 2017; Stein & Stein, 2013). The failure to properly take account of the full range of uncertainty can pose significant problems for risk management, from the flawed safety measures at the Fukushima power plant, to the reliance on short‐term earthquake prediction models (reportedly at the expense of mitigation efforts) in modern China. This will not be news to many within the hazard modeling community––indeed, there has been significant progress in methods for characterizing uncertainty in recent years, making now a good time to take stock of developments and explore areas for potential improvement.

My scope is restricted to hazard models designed to inform decision making, rather than theoretical models. This distinction matters for two main reasons. The first is that the goals of both modeling communities differ, and by extension, the appropriate forms of uncertainty analysis will vary. Theoretical models aim to explain the workings of the natural world––to capture the fundamental physical processes at play––whereas models designed to inform decision making are primarily concerned with making informative, good predictions.^1^ As a result, it may be unusual to see formalized approaches to expert elicitation used in basic research, as science advances by argument and evidence, rather than by votes, whereas such procedures may prove to be a useful way to inform decisionmakers of the range of expert (dis)agreement on a given matter. Similarly, a formal characterization of the full range of model and parameter uncertainty may help decisionmakers understand how robust their choices are to the range of known unknowns, but be quite superfluous for theoretical modeling. A second reason the distinction matters is that political pressures––perceived or real––may play a more significant role in the generation of science for policy applications, particularly when this takes place within government institutions. I define a model as a simplified representation of a target activity or system, and conceive of uncertainty in the broad sense of incomplete or imperfect knowledge (SRA, 2018). I distinguish three key types of uncertainty (Hill et al., 2013; Linkov & Burmistrov, 2003; Parker, 2013):

1) Parameter uncertainty: uncertainty surrounding the correct value of model parameters;
2) Input uncertainty: stemming from incomplete knowledge of the initial state of the system (initial conditions; boundary conditions);
3) Structural uncertainty: the inability of the model to represent the target system, even if the correct inputs and parameters are known (e.g., relating to the form of modeling equations and how they should be solved computationally).

With these clarifications out of the way, I ask what, then, are the key sources of uncertainties in models of earthquake‐induced hazards? How are they currently handled (or neglected), and with what practical implications? How might we do better? In exploring such questions, this article aims to:

1) Provide a thematic overview of key sources of uncertainty in hazard models of earthquakes, tsunamis, and landslides;
2) Critically evaluate current methods for characterizing those uncertainties;
3) Identify, where relevant, strategies for more rigorous, transparent handling of uncertainty.

The article's approach is necessarily schematic, emphasizing key themes, rather than providing a detailed survey of the literature. The analysis is organized by hazard type, followed by an overarching discussion.

## 2 PROBABILISTIC SEISMIC HAZARD ASSESSMENT

Earthquake forecasting methods date back to antiquity. Aristotle developed one of the earliest theories of earthquakes––based on the notion that they were driven by wind trapped beneath the earth's surface––and from this account identified a series of early warning signals, for example, anomalous subterranean gas emissions; changes in animal behavior; clouding and change in taste of well water (Missiakoulis, 2008; See, 1907). Although his theory is long defunct, the idea that there are identifiable precursors of an impending earthquake lives on. Indeed, the late 1960s–1970s saw increasing optimism within the scientific community that earthquakes could be predicted days or even hours in advance, although this later transformed into skepticism as purported precursors could not be reliably validated (Geller, 2011; Geller, Jackson, Kagan, & Mulargia, 1997). While the majority of the seismology community soon came to view the short‐term prediction of large earthquakes as either currently unrealistic or outright pseudoscience, it remained a respectable, government sanctioned line of research that fed into decision support systems in China and Japan (Chen & Wang, 2010; Geller, 2011).^2^ What might account for this?

The long shelf‐life of China's prediction program has been attributed to political and ideological drivers. Following the apparent prediction of the (1976) Haicheng earthquake––the only successful prediction in recorded history––the program became a source of national pride at a time when Chinese scientists were isolated internationally (Fan, 2007), and its putative success was portrayed as demonstrating the superiority of the socialist system and the “victory of the proletarian cultural revolution” (Cha, 1976). Moreover, precursor hunting was coherent with core tenets of Maoist thought, such as the ambition of mastering nature via prediction and control, as well as representing a critique of “elite” science combined with the veneration of amateur involvement (precursors such as variations in animal behavior, groundwater levels, etc. being accessible to untrained observers) (Cha, 1976; Fan, 2007). The Division Head of China's National Earthquake Bureau attributed skepticism about earthquake prediction to antirevolutionary prejudices, and claimed that the previous regime's concern with earthquakes was restricted to the opportunity they presented to “loan money with usurious interest to the hard pressed people” (Cha, 1976). Finally, the prediction program reflected a traditional epistemology––famously embodied in Chinese medicine––that mechanistic knowledge is unnecessary for reliable prediction (Fan, 2007). Indeed, it was only the failure to foresee the catastrophic (2008) Wenchuan earthquake that marked a decisive shift within China from short‐term forecasting efforts toward an emphasis on risk mitigation (Chen & Wang, 2010). In Japan, similarly, the Meteorological Agency has been legally bound since 1978 to identify and evaluate precursors indicating that the “Tokai earthquake” will occur imminently (Geller, 2011). Such a law––which reportedly became moribund in late 2017––is without modern parallel (ibid.). Geller (2011) attributed its entrenchment to the innate conservatism of Japanese scholarly and media sectors, the co‐opting of potential critics via appointments to prestigious advisory boards, and the fact that once practices are sanctioned by law, they acquire a certain path dependency.

There is one general lesson and one question that I wish to draw from the above examples. The general lesson is that while we are used to thinking of how policymakers act in the face of uncertain scientific information, state institutions may actively construct ignorance through endorsing modeling approaches that encode assumptions that are scientifically questionable, yet that are politically or ideologically attractive. The question is whether an *unrealistic* faith in prediction models can undermine resilience by creating a misleading sense of security that sidelines risk mitigation efforts (Chen & Wang, 2010). The evidence supporting this is based on historical accounts. Chen (member of the Chinese Earthquake Administration) and Wang (2010) use archival data to trace out the historical evolution of China's earthquake prediction program. They recount that organized efforts of earthquake prediction began in 1966 before climaxing in the seeming prediction of the Haicheng earthquake (1976). During this period, the Chinese leadership viewed prediction as a “faster and cheaper solution than improving the quality of buildings” (Chen & Wang, 2010). They conclude that the resources directed toward the prediction program “certainly” diluted the focus on mitigation at least until the passing of the Earthquake Act in 1998, and speculate that the associated false sense of security associated with the promotion of prediction “success stories” may be partially responsible for the lax enforcement of mitigation measures required by the aforementioned legislation. In contrast, Japan's history of substantial investments in mitigating earthquake hazards implies that the Tokai prediction program has been relatively benign in this respect, although some have speculated that the program distracted attention from other risk‐prone regions of the country (Nöggerath, Geller, & Gusiakov, 2011). The hypothesis that a focus on prediction efforts diverts attention from mitigation efforts appears to be relatively commonly held among seismologists (Joffe, Rossetto, Bradley, & O'Connor, 2018). The underlying assumption appears to be that governments tend to allocate a relatively fixed, aggregate budget for earthquake prediction and preparedness, and so resources directed toward the former by necessity come from the latter. Whether this is a plausible assumption depends on institutional funding mechanisms. For example, in the modern United States, seismic risk mitigation efforts are essentially paid for by developers who must adhere to federal safety regulations, while research supporting the development and implementation of these regulations is largely funded by the Engineering Directorate of the NSF. Would an increase in funding for short‐term prediction efforts (presumably from the Geosciences Directorate) necessarily imply a reduction in research in infrastructure resilience (typically funded by the Engineering directorate)?^3^ Stated so bluntly it may seem implausible, although the historical record provides some pause for thought. Prior to the 1977 passing of the Earthquake Hazard Reduction Act in the U.S. Senate, a panel of scientists and engineers were tasked with considering the extent to which the proposed $50 million a year program “should focus on prediction, as opposed to efforts to mitigate earthquake risk” (Hough, 2016). Although this supports the hypothesis that an *unrealistic* faith in prediction models can undermine resilience, in practice little of the funds were allocated toward prediction (Hough, 2016) as optimism surrounding its feasibility declined.

Few seismologists now believe that precursors can be reliably used to predict the occurrence of large earthquakes. Longer‐term forecasting efforts––in the form of probabilistic seismic hazard assessment (PSHA)––now represent the “first line of defense with respect to mitigating earthquake risk” (Field & Milner, 2018). Within PSHA, the distribution of seismic hazard is estimated based on: (1) the location of the site with respect to known or assumed earthquake sources, (2) the assumed recurrence behavior of these earthquake sources, and (3) the computed ground motion for the earthquakes at the given site (e.g., peak ground acceleration) (Cornell, 1968; Stirling, 2014). PSHA outputs include estimates of the maximum expected ground shaking with a given probability over a specified period of time (Budnitz et al., 1998). PSHA is challenging to conduct and evaluate, given the long recurrence periods, chaotic behavior, and difficulties of observing underlying processes. While the basic theory of plate tectonics is long‐established, uncertainty surrounds how plate motion is actually released during earthquakes (Stein, Geller, & Liu, 2012). Moreover, controversy surrounds the distribution of earthquake recurrence intervals, with the traditional choice being between a probability density function that views the recurrence of large earthquake as time‐independent (a Poisson process), and a time‐dependent recurrence model that presumes a quasi‐periodic return period (the “seismic gap”model) (Field et al., 2017). Applications of PSHA also face significant parameter uncertainty, for example, relating to the locations of faults, their activity, how rapidly they accumulate strain, and how that strain will be released (Stein et al., 2012).

Until the past decade or so, the development of PSHA models proceeded largely in the absence of systematic attempts at validation (Stirling, 2014). However, initiatives, such as regional earthquake likelihood models (RELM) and collaboratory for the study of earthquake predictability (CSEP), have emerged to correct this discrepancy (Field, 2007; Jordan, 2006). These initiatives have found forecasting models to be increasingly skillful and reliable (Schorlemmer et al., 2010; Zechar et al., 2013). Given the exponential distribution of earthquake magnitudes, relatively small magnitude earthquakes constitute the majority of the test data (Marzocchi & Zechar, 2011). As such, it is difficult to directly evaluate the predictive accuracy of PSHA models in relation to large earthquakes. However, several recent large earthquakes occurred in regions previously characterized by PSHA analyses as low hazard (Stein et al., 2012). How should these seemingly conflicting lines of evidence be reconciled or balanced? If one views system‐size earthquakes as fundamentally the same as their smaller counterparts, then the positive findings of the RELM and CSEP initiatives should be decisive. However, on this former issue, the seismology community is split (Marzocchi & Zechar, 2011).

Nevertheless, even if one views large earthquakes as fundamentally different from their smaller counterparts, then it is by no means clear that the recent occurrence of large earthquakes in low hazard areas is an indictment of the PSHA approach. Such claims have been forwarded in the literature (e.g., Stein & Stein, 2013). My own view is that these claims amount to “naive falsificationism,” wherein a discrepancy between a model or hypotheses and observation(s) is interpreted as grounds for rejecting said model, and in extreme instances, as discrediting the underlying methodology. In practice, the aforementioned discrepancy may be down to a host of other factors, such as errors in input data, coding, or even just random chance or the use of inappropriate evaluation statistics.^4^ Even if the discrepancy stems from structural error, then once more this is insufficient ground for rejecting the model(s) or abandoning PSHA, at least if we are to take Box's (1979) maxim at all seriously. Indeed, on close inspection, many of the more hostile critiques of PSHA come from a strict frequentist perspective, wherein PSHA outputs are variously described as “statistical nonsense,” untestable (Mulargia, Visconti, & Geller, 2018), and the outcome of “opinions and ad hoc choices” (Stark, 2017). These claims are unlikely to hold much water with PSHA practitioners, nor indeed with the broader risk assessment community, given that risk and decision analysis has its roots in Bayesian reasoning (Kaplan & Garrick, 1981). Below, I focus on how uncertainty has been handled in the landmark Uniform California Earthquake Rupture Forecast (UCERF) assessments, which provide official earthquake rupture forecasts for California.

UCERF, in common with state‐of‐the‐art PSHA applications, characterizes model uncertainty via an ensemble of alternative models that are consistent with current knowledge. Historic data––particularly for high‐intensity, low‐frequency earthquakes––are too sparse to weight each model with reference to conventional measures of fit (e.g., Bayesian information criteria) (Marzocchi & Jordan, 2014). And so in practice, model uncertainty in PSHA is represented via logic trees (sometimes involving several thousand alternative paths), where each branch represents a viable alternative model choice or assumption (e.g., Field et al., 2017). Probabilities are assigned to each branch, and these are typically intended to reflect the perceived probability that the model represents “the true state of nature” (Field et al., 2009), or in more recent interpretations, that it is the best available choice (Field et al., 2017). Past UCERF exercises faced criticism for the basis on which probabilities were assigned. For example, Page and Carlson (2006) criticized the use of data‐availability as a criterion for assigning weights to alternative earthquake‐recurrence models in the first UCERF exercise. They argued that the volume of data available is orthogonal to the question of which model is (more likely to be) true, and that as a result, such a weighting scheme can introduce bias (Page & Carlson, 2006). However, in recent years, analysts have come to favor the interpretation that the probabilities are measures of confidence that the model is the best available choice, as opposed to the probability that it is true. Under such an interpretation, the use of data‐availability as a criterion for assigning weights seems to be a pragmatic choice. The most recent UCERF exercise provides more evidence on the criteria underpinning the probability assignments (e.g., Parsons et al., 2013). The criteria appear to fit within the broad class of epistemic values––those values that promote the acquisition of true beliefs (Goldman, 1999)––including notions such as fit to data sets and consistency with background theory (Parsons et al., 2013). Some philosophers argue, however, that risk assessors should also consider nonepistemic values when, inter alia, assigning probabilities to alternative models (e.g., Douglas, 2000). These nonepistemic values refer to social and ethical considerations, such as whether to be precautionary when making methodological choices, and how conflicting goals (e.g., human life vs. economic cost) should be weighed in determining how precautionary to be. My own view is that these normative questions should not influence risk or hazard assessment as this would undermine their objectivity. Instead, they should be addressed within decision analysis frameworks, as these provide formal, explicit processes for expressing values and characterizing tradeoffs (MacGillivray, 2019; but see Hicks, Magnus, & Wright, 2020, for a response).

A separate critique of the logic tree approach––as undertaken in UCERF and PSHA more broadly––has focused on what is argued to be the implicit assumption that the models represented at each tree branch are mutually exclusive and completely exhaustive (MECE) (Page & Carlson, 2006). This MECE criterion appears to be violated in PSHA practice. For example, in UCERF2, important drivers of seismic hazard––such as those relating to fault‐to‐fault ruptures and earthquake‐clustering effects––were not well enough understood to allow for formal representation (Field et al., 2009). More generally, logic tree branches within PSHA are not thought of as exhausting all possibilities (Marzocchi, Taroni, & Selva, 2015). What are the conceptual and practical implications of seemingly violating MECE? Marzocchi and Jordan (2014) argue that there is no such violation as long as the probabilities are interpreted in terms of the “best among a set of available models,” as opposed to the “true model.” Parker (2013) has similarly argued that the latter interpretation is naïve in relation to climate model ensembles. Put simply, Box's (1979) maxim makes it difficult to entertain the idea of an ensemble that spans “the full range of current uncertainty about model structure.” What are the implications of this? My own view is that PSHA outputs should be interpreted as conditional probability estimates (see also Hansson & Aven, 2014), in other words, they are conditional on a knowledge base that may be more or less strong (Aven, 2013a,b; Kaplan & Garrick, 1981). As a corollary, the strength of this knowledge base should be communicated to decisionmakers. Measures of confidence in PSHA outputs can be conveyed, for example, through assertions of the degree to which assessors expect to update their probabilities in the face of new data (Lehner, Laskey, & Dubois, 1996). This is now a standard practice in the IPCC's climate change assessments (Mastrandrea et al., 2011). Adopting such a practice would respond to concerns raised by some analysts that decisionmakers tend to view PSHA outputs as a “unique and precise representation of the reality,” a tendency which “caused considerable confusion among decision makers when large swings of the mean hazard may occur in the wake of model changes and updates” (Lee, Graf, & Hu, 2018).

I turn now to the process for eliciting logic branch probabilities. In UCERF2, alternative branches were assigned equal probabilities in the absence of “clear evidence to favor a given one over the other,” and where such evidence exists, the probability values were assigned through a “consensus‐building process” (Field et al., 2009). In the most recent UCERF3 exercise, branch probabilities were in one application assigned on the basis of an “informal poll taken among those in [workshop] attendance”(Field et al., 2015), while in other instances, ad hoc special committees were established for this purpose. These descriptions suggest that elements of both behavioral and mathematical approaches to eliciting and aggregating expert judgments were relied upon. This may seem like a curious combination; however, the purposes of expert elicitation for PSHA are somewhat nonstandard. That is to say that they are not designed to capture and aggregate the probability judgments residing in the experts’ heads, but rather to determine the “center, body, and range of technical interpretations” of the informed technical community (Budnitz et al., 1997). A large literature emphasizes that such process design features can play a significant influence in shaping the outcomes of expert elicitation, including within PSHA (e.g., Runge, Scherbaum, Curtis, & Riggelsen, 2013; Scherbaum & Kuehn, 2011). Some scholars argue that consensus estimates produced by behavioral methods (e.g., Delphi) reflect strong group pressures for conformity rather than genuine agreement (Morgan, 2014; Woudenberg, 1991). Another stock concern is that even experts tend to be poor at producing probability judgments in the absence of a formal, structured approach to elicitation and aggregation (Scherbaum & Kuehn, 2011). Whether these concerns have any bearing on recent UCERF exercises is unclear, given that the full details of the elicitation and aggregation procedures do not appear to be in the public domain. However, what is interesting is that, if one interprets (1) the selection of process design features as a modeling choice with testable implications, and (2) that recent initiatives, such as RELM and CSEP, provide a framework for evaluating these implications, then (3) there is, in principle, the possibility to empirically determine which process‐designs will generate more reliable, skillful PSHA estimates.

## 3 TSUNAMI HAZARD ANALYSIS

Tsunamis––long waves of limited steepness generated by geophysical events, including earthquakes, submarine landslides, volcanic eruptions, and asteroid impacts––are among the most devastating and difficult to predict hazards (Synolakis & Bernard, 2006). Tsunami hazard analysis is broadly concerned with simulating wave initiation, propagation, runup, and inundation, although models may focus on distinct timescales or components of the hazard‐chain. A useful distinction is that between short‐term forecasts in support of early warning systems, and long‐term forecasts, such as inundation estimates in support of site‐specific hazard evaluations or hazard mapping (Titov et al., 2011). Early warning systems depend upon models that simulate wave propagation and runup conditional upon knowledge about the triggering event (e.g., macroscopic earthquake parameters) and wave evolution (e.g., from deep‐sea tsunameters). Site‐specific hazard evaluations––for example of nuclear power plants––on the other hand condition their assessments on knowledge of the seismic hazard profile of the region, and may involve simulations of the impact of waves upon the structure itself (i.e., the consequences of the hazard). Tsunami risk maps similarly condition upon the seismic hazard profile to estimate the probability of inundation, perhaps combined with an approximate measure of consequences (e.g., tsunami loss curve), to inform land use planning and mitigation measures.

Tsunami hazard modeling was pioneered by the Japanese, with the first early warning system developed in 1941, based on an empirical assessment of the relations between earthquake amplitude and distance from source (Bernard & Titov, 2015). However, the size of tsunami generated by an earthquake is a function of several factors in addition to earthquake magnitude, including source mechanism, fault rupture velocity, hypocentral depth, and water depth within the source region (Gusiakov, 2009). A particular problem is posed by tsunami‐earthquakes, defined as seafloor motions that generate wave amplitudes far greater than would be expected based on earthquake magnitudes alone. As such, seismic intensity is an imperfect proxy for tsunami hazard––indeed, tsunami amplitudes can differ by a factor of up to 60 for earthquakes of the same magnitude (Gusiakov, 2009)––and relying on it within either formal or informal warning systems can be disastrous.^5^ Modern early warning systems synthesize the knowledge of macroscopic earthquake parameters with data from tsunameters to provide more reliable predictions, although uncertainty remains. For example, the 2011 Tohoku tsunami was severely underestimated (3–6 meters vs. 10+ meters) due to incorrect initial estimates about the magnitude of the earthquake (7.9 vs. true value of 9.0) (Goda & Abilova, 2016). Forecasting is even more challenging where coseismic hazards are involved (e.g., underwater landslides) and for tsunamis generated by nonseismic events (e.g., volcanic eruptions) for which no precursors may be available. (Gusiakov, 2015). These challenges apply to early warning systems, and to long‐term site‐specific evaluations and hazard mapping efforts, which I focus on below.

While the mathematics governing tsunami motions––the Navier Stokes equations––have been known for over 150 years, they are notoriously difficult to solve, necessitating a host of approximations and idealizations (e.g., the elimination of viscous stresses), and numerical rather than analytical solutions (Synolakis & Bernard, 2006). Recent decades have seen significant improvements in modeling capabilities, driven by the widespread deployment of tsunameters, more detailed bathymetric maps, systematic field surveys in the aftermath of major tsunamis (e.g., the 1983 Japan Sea and 1993 Hokkaido Nansei‐Oki tsunamis), improved computational capacities, and an international multimodel benchmarking exercise, which served as the basis for the evaluation and improvement of simulation techniques (Kânoğlu, Titov, Bernard, & Synolakis, 2015; Shuto & Fujima, 2009). A development worth highlighting is that the influence of near‐shore bathymetry and onshore topography––including human modifications and land‐use––appears to play a more significant role in shaping inundation and runup than previously thought (Kânoğlu et al., 2015; Synolakis & Bernard, 2006).^6^ I highlight this because it remains a widespread practice to interrupt the calculation of tsunami simulations at some arbitrary offshore location, from which inundation is inferred (Synolakis & Bernard, 2006; Synolakis & Kânoğlu, 2015). Indeed, one of the questionable aspects of the pre‐event hazard analysis conducted for the Fukushima power plant was that they adopted this threshold methodology, with the consequence that they seemingly did not consider the possibility of overland inundation (Synolakis & Kânoğlu, 2015). However, the dominant source of uncertainty in long‐term tsunami hazard evaluations surrounds the initial conditions (i.e., the triggering event) (England, Howell, Jackson, & Synolakis, 2015).

One (precautionary) way of handling this uncertainty is to base hazard analyses on the “maximum probable tsunami.” How are these identified? Japanese regulatory guidance sets out the following standard procedure: identify the largest historic tsunami for which reliable data exist; identify the tsunami that would stem from the largest earthquake that could occur conditional on current scientific knowledge; then select the tsunami with the higher water level on the coast (Shuto & Fujima, 2009). It is important to emphasize that rule‐based approaches to hazard assessment, whether in the form of explicit guidelines or semiautomated modeling procedures, do not obviate the need for expert judgment. As a cautionary example, the calculation of the maximum probable tsunami pre‐Fukushima contained basic methodological flaws “which almost nobody experienced in tsunami engineering would have made” (Goda, Mai, Yasuda, & Mori, 2014), resulting in plant safety design features that were far from adequate (Synolakis & Kânoğlu, 2015). An alternative to “precautionary” analyses is to use PSHA outputs as inputs to tsunami hazard analyses. Goda et al. (2014) use an ensemble of earthquake source models as a means of reflecting some of the underlying uncertainties. Particular challenges are posed by tsunami‐earthquakes^7^ and nonseismic tsunami triggers (e.g., submarine landslides and volcanic eruptions). The limited physical theory and empirical evidence relating to these triggers means that there is no reliable basis for assigning probabilities to them (Synolakis & Kânoğlu, 2015; Tappin, 2018), making them challenging to take account of in hazard analysis.

Parametric uncertainty, on the other hand, is widely handled through sensitivity analysis in tsunami hazard assessment. Conventionally, this is done through varying one parameter or input value at a time over an arbitrarily limited space (Goda et al., 2014), but this is strictly speaking inadvisable for correlated sources of error. Methods of global sensitivity analysis (GSA) have recently been applied in tsunami forecasting, although are computationally demanding (Goda, Yasuda, Mori, & Mai, 2015). I return to this topic in relation to debris flow modeling.

## 4 DEBRIS FLOW MODELS

Earthquakes play a significant role in destabilizing slopes, creating coseismic landslide hazards, and enhancing the probability and volume of subsequent rainfall‐induced debris flows. Debris flows, which I focus upon, carry the potential for large‐scale destruction of infrastructure as well as posing a substantial threat to human lives (Nolde & Joe, 2013). Debris flow hazard assessments ask two distinct questions: where and when will debris flows initiate, and how large will they be (initiation); and what speed will they travel, and which downstream areas will be affected (propagation) (Iverson, 2014). Physical and empirical models have been deployed for both tasks. Physical models carry the benefits of being rooted in basic principles of the conservation of mass and momentum (and so are transportable across space and time); can explicitly represent interactions between debris flows and the surrounding environment (e.g., redirection/overtopping in the face of an obstacle such as check dam); and allow for (counterfactual) reasoning about potential risk mitigation interventions (e.g., slope stability measures) on the hazard profile (Iverson, 2014; Iverson, George, & Logan, 2016). However, in practice, their implementation for the purposes of decision support faces myriad challenges (Almeida, Holcombe, Pianosi, & Wagener, 2017; Iverson, 2014). There are multiple plausible representations of debris flow initiation and propagation processes––each with their own omissions, idealizations, and assumptions––and neither field nor experimental data provide decisive tests between them (Bennett, Molnar, McArdell, & Burlando, 2014; Iverson, 2014). Parameter uncertainty in physical models is often handled by sensitivity analysis, wherein a range of values for initial conditions and material properties (e.g., slope stability) are selected and used to compute an associated range of potential outcomes. However, the sensitivity analysis is typically local, meaning that only a limited range of parameter variation is explored around a base value or reference case (Rohmer, 2014). This method is efficient and so is particularly useful for complex models; however, extrapolation of the results beyond these small perturbations to cover the entire feasible space of parameter variation is only valid where nonlinearity in the input–output relations can be ignored (Rohmer, 2014). Moreover, parameters are typically varied one‐at‐a‐time, meaning that uncertainties stemming from any interactions (i.e., correlated parameters) are not accounted for (Rohmer, 2014).

Given the above challenges, empirical models play a dominant role in debris flow hazard assessment, particularly in early warning systems, but also to inform longer‐term decisions, such as land‐use zoning. I focus here on the use of debris flow initiation models (rather than propagation), which correlate the intensity and duration of rainfall events^8^ with the (probability) of debris flow initiation to derive rainfall intensity‐duration thresholds (e.g., Liu et al., 2016). Standard curve‐fitting techniques mean that they often have reasonable measures of fit to data sets. However, these measures should be interpreted cautiously: while the models contain a limited number of free parameters (reducing the risk of overfitting), discretionary choices of functional form, of which rainfall index to use, and of which time period to use for calibration mean that significant degrees of freedom remain (Osanai, Shimizu, Kuramoto, Kojima, & Noro, 2010). Model performance out of sample, particularly when extrapolated across time or location, can fall significantly (Huang, van Asch, Wang, & Li, 2019; Papa, Medina, Ciervo, & Bateman, 2013). This is due to varying soil, land‐cover and lithological conditions, as well as a typically scarce record of reliably reported events (Fan et al., 2019). As such, the application of empirical models in emergency warning systems is often restricted to catchments where the model was calibrated, although more ambitious efforts attempt to derive rainfall intensity‐duration thresholds for broader regions characterized by specific combinations of hydrology, topography, and geology (Alfieri, Salamon, Pappenberger, Wetterhall, & Thielen, 2012; Iverson, 2014).

Empirical debris flow models typically do not explicitly incorporate uncertainty, instead addressing it through attaching safety factors to the thresholds at which early warnings are triggered. To my knowledge, the establishment of such thresholds has not been informed by an explicit, systematic consideration of the relative costs of false positives and false negatives, for example, within a decision‐theoretic framework. Such approaches have been developed in relation to weather forecasting (e.g., Economou, Stephenson, Rougier, Neal, & Mylne, 2016; Roulston & Smith, 2004). More on this later. A key source of uncertainty is the implicit assumption that the parameter values are relatively stable feature of the world (i.e., ergodicity). When any of the relevant background variables change––for example, where a check dam is introduced to a gully, or where land use changes have influenced drainage systems––parameter stability can no longer be assumed. One way to handle this is to recalibrate the model following significant changes, for example, the introduction of a check dam, although of course this involves the classic bias‐variance tradeoff. Moreover, violations of the ergodicity assumption are not always clear‐cut. For example, the ongoing redistribution of sediment across catchments in Sichuan province––over a decade after the Wenchuan (2008) earthquake mobilized approximately 3 km^3^ of material across the Longmenshan Fault (Li et al., 2014)––has continually altered patterns in the location and frequency of debris flows (Hales et al., 2017). The implication is that debris flow initiation probabilities are continually evolving in the real world. As a consequence, a recent review concluded that physically based models and geotechnical investigations are preferable for deriving landslide‐triggering rainfall thresholds following major earthquakes (Fan et al., 2019).

A concern sometimes raised with the approach to debris flow risk management adopted in hazard‐prone mountainous regions of Western China (particularly in Sichuan and Gansu Province)––characterized by a reliance on emergency warning systems and check dams––is that it may not only be ineffective, but may also be providing a (misleading) sense of security that encourages settlement in highly hazardous areas (e.g., Xiong et al., 2016). For example, the current engineering design standard for check dams is premised upon the assumption that debris flow discharges are a linear function of precipitation, and as a result can underestimate the volume of debris flows by up to an order of magnitude (Horton, Hales, Ouyang, & Fan, 2019; Xu, Zhang, Li, & Van Asch, 2012). Indeed, Chen, Cui, You, Chen, and Li (2015) reported that not only did such control measures fail to contain peak flow discharges in Wenjia Gully, Sichuan, but that their destruction served to amplify the scale of the debris flow. Xiong et al. (2016) report similar findings from Sanyanyu, Gansu Province, where the collapse of check dams amplified a debris flow that subsequently killed 1,756 people. These examples raise the question of whether Gansu and Sichuan's approach to debris flow risk management is leading to something analogous to the “levee effect.”

## 5 SYNTHESIS AND CONCLUSIONS

Modeling earthquake‐induced hazards involves multiple, nontrivial sources of uncertainty. Initial and boundary conditions are often poorly constrained; model structures contain omissions, approximations, and idealizations whose implications are challenging to evaluate; and parameter values often cannot be reliably determined by theory or empirics. This, combined with the nonlinear and chaotic nature of many key geophysical processes, means that *unconditional*, short‐term predictions of hazardous events are infeasible at present and perhaps impossible in principle. None of this is to suggest that hazard models are not useful for decision making. Indeed, short‐term predictions conditional on knowledge of triggering events have proven useful for emergency management, for example, in tsunami early warning systems, and operational earthquake forecasting efforts have shown promise in New Zealand, Italy, and the United States for simulating aftershock sequences (Harte, 2019; Milner, Field, Savran, Page, & Jordan, 2020). Longer‐term hazard forecasts have been similarly valuable for planning purposes (e.g., seismic hazard assessments of nuclear power plants and critical infrastructure), and in many regions remain the first line of defense against earthquakes, tsunamis, and debris flows.

An apparent trend across our hazard domains is the progression from empirical to more physically based hazard models. Physical models in principle provide greater epistemic confidence. They are more readily generalizable across time and space, allow users to pose a range of “what‐if” questions (e.g., the effects of potential risk mitigation options), and can explicitly represent key interactions between hazards and boundary conditions (e.g., the influence of nearshore topography on tsunami inundation flows). However, empirical methods remain very useful in many contexts. Indeed, as Field (2019) suggests, the question of physical versus empirical models is something of a red herring, as “whether any model is reliable or trustworthy depends entirely on what questions we are asking of it,” and, I would add, on whether the key sources of uncertainty have been adequately characterized.

Uncertainty in model inputs and parameter values is routinely handled by some form of sensitivity analysis. However, typically only a limited range of variation is explored within debris flow and tsunami modeling, and potential interactions are not commonly considered (Goda et al., 2014; Rohmer, 2014). GSA is a promising method for covering the full space of parameter uncertainty and considering correlated sources of errors, and moreover does not require the specification of (often arbitrarily chosen) probability distributions (Saltelli et al., 2008). GSA is computationally demanding for complex models; in such cases, emulators may be a useful compromise (Coutts & Yokomizo, 2014; Rohmer & Foerster, 2011). My focus, however, has been on structural uncertainty.

Box's aphorism that all models are wrong (but some useful) implies that characterizations of uncertainty which are conditional on the truth of a model are insufficient. Broad consensus on underlying physical theories (e.g., plate tectonics) can rest alongside significant uncertainty on how to formally represent hazard processes and implement them in numerical models. Notwithstanding the significance of this uncertainty, I have argued that some of the more hostile criticisms of PSHA should be discounted as they rest upon “naive falsificationism” and a strong distaste for Bayesian subjectivity. Logic trees (e.g., in PSHA) are one way of representing structural uncertainty through assigning probability values to alternative assumptions or model choices. These probabilities are typically assigned via expert elicitation, although there is some debate within the field as to which process should be adopted for this purpose. I have suggested that the design of the expect elicitation procedure be viewed as a modeling choice with testable implications, and that model evaluation initiatives, such as RELM and CSEP, may offer a framework for determining which process‐designs produce more reliable, skillful PSHA estimates.

PSHA logic trees often involve several thousand branches, which in practice must be sampled prior to estimating the hazard measure of interest, given limits on computing power. See Porter, Field, and Milner (2017) and Marzocchi et al. (2015) for rigorous methods for doing so. Only under the strong assumption of the MECE criterion will logic tree or multimodel ensemble outputs reflect the full range of structural uncertainty (Morgan & Henrion, 1990; Saltelli et al., 2008). I have argued that this will typically be a naïve assumption, and that as a consequence, such outputs should be interpreted as lower‐bound estimates of structural uncertainty. As a corollary, measures of confidence in these risk assessment outputs should be conveyed to decisionmakers, for example, through assertions of the degree to which assessors expect to update their probabilities in the face of new data (Lehner et al., 1996). A relatively simple, informal approach for doing so has been adopted within the IPCC's climate change assessments (Mastrandrea et al., 2011), and may prove useful in PSHA. For more formal and potentially transferable approaches to handling uncertainty in multimodel ensembles within climate science, see Rougier, Goldstein, and House (2013) and Rougier (2007).

An unfortunate way of handling uncertainty is to neglect to characterize and communicate it, typically motivated by the questionable belief that decisionmakers desire precise, definitive analysis outputs (Stirling, 2010). In extreme cases, modeling frameworks may become entrenched in institutional decision‐making processes––for example, because they align with prevalent political ideologies––despite encoding assumptions that are scientifically questionable (e.g., China's short‐term earthquake prediction program). Alternatively, hazard models may produce “uncomfortable knowledge,” in the sense that they are perceived to dictate politically unpalatable choices, and as a result may be discredited or neglected (MacGillivray & Richards, 2015; Rayner, 2012). Although the natural response to this may be to advocate the insulation of hazard analysis from social or political pressures, a rough consensus exists that risk management is better served by the integration rather than strict separation of hazard analysis and decision making (NRC, 1994).

Formal decision‐analytic frameworks can be useful for this purpose, both standard and nonstandard. The decision theoretic approach––based on Savage's theory of subjective expected utility––is often viewed as the “gold standard”; however, its normative status cannot be assumed to hold beyond idealized conditions, which may be far from the reality of most geophysical applications (Freedman, 1997). In such situations, inexact methods of problem‐solving may be more defensible (Cox, 2012; Heal & Millner, 2014; Jaynes, 2003; Lempert & Collins, 2007). Indeed, as discussed, precautionary approaches have been adopted within debris flow and tsunami early warning systems (e.g., in setting emergency warning thresholds), as well as within site‐specific tsunami hazard evaluations. However, the relative costs of false positives and false negatives have not been explicitly considered in such applications. Such informal approaches can lead to the adoption of a series of precautionary assumptions that are individually reasonable but collectively implausible (Hill et al., 2013). For example, tsunami early warning systems that err substantially on the side of “safety” have not only led to widespread economic disruption but also in some cases fostered a climate of public skepticism, with the result that (genuine) evacuation warnings were neglected, leading in some cases to substantial casualties (Bernard & Titov, 2015; Plümper, Flores, & Neumayer, 2017). As such, I suggested that the development of early warning thresholds might be informed by an explicit decision analysis (see, e.g., Economou et al., 2016). The public skepticism mentioned above, stemming from the experience of false alarms, implies that such a decision analysis should be informed by an assessment of the likelihood of compliance, including a consideration of how compliance evolves in light of experience with early warning systems. For a framework for doing so, see Roulston and Smith (2004). More broadly, this reminds us that the relative costs and benefits of a given risk mitigation measure (e.g., a check dam) are often contingent on assumptions or forecasts about how individuals and communities will behave in light of said measure (e.g., by populating a debris flow prone channel). Indeed, the cases of Wenjia Gully, Sichuan, and Sanyanyu, Gansu Province, led me to speculate whether the approach to debris flow risk management adopted in hazard‐prone mountainous regions of Western China is leading to something analogous to the “levee effect.” Nevertheless, in situations where behavioral responses to mitigation measures are thought to have a significant influence on outcomes, and where those responses are challenging to determine *a priori*, then robust decision‐making frameworks (Lempert & Collins, 2007) or sequential strategies (Simpson et al., 2016) may prove to be a more defensible approach to risk management.

## Footnotes

1 By *informative* predictions, I mean predictions that are relevant to some real‐world decision problem. By good *predictions*, I mean reliable statements about a (as yet unobserved) feature of the world. Some prefer to distinguish predictions from forecasts and counterfactual analyses, although I view the latter two as a subset of predictions, with predictions simply being statements about the world that are in principle testable.

2 Pure research on this topic continues elsewhere in the world.

3 I owe this point to a reviewer.

4 I owe the latter point to a reviewer.

5 Perhaps the classic illustration is the Meiji Great Sanriku tsunami earthquake (1896), which struck northeastern Japan with run‐up heights (up to 38 m) far in excess of what would have been expected based on the weak ground shaking (Shuto & Fijima, 2009). The lack of precursors meant no evacuation, and a loss of life of upward of 22,000. More recently, residents of Papua New Guinea neglected to evacuate in response to an offshore 7.1 magnitude earthquake on the grounds that it was not particularly large (Monastersky, 2012). The earthquake triggered an underwater landslide, and the subsequent tsunami cost 2,000 lives.

6 For example, field observations following the 1994 East Java and 1996 Peruvian tsunamis further support the idea that extreme nearshore features play a significant role in shaping the distribution of run‐up levels even across relatively open and straight coastlines. Moreover, estimates of run‐up values generated from threshold approaches differ from those that explicitly account for near‐shore bathymetry and topography by a factor of two or more (Kânoğlu, Titov, Bernard, & Synolakis, 2015).

7 Recall that these are not simply earthquakes induced by tsunamis, but seafloor motions that generate wave amplitudes far greater than would be expected based on earthquake magnitudes alone.

8 The primary trigger for debris flows.
